# The quality of turning in Parkinson’s disease: a compensatory strategy to prevent postural instability?

**DOI:** 10.1186/s12984-016-0147-4

**Published:** 2016-04-19

**Authors:** Sabato Mellone, Martina Mancini, Laurie A. King, Fay B. Horak, Lorenzo Chiari

**Affiliations:** Department of Electrical, Electronic and Information Engineering “Guglielmo Marconi”, University of Bologna, Bologna, Italy; Department of Neurology, School of Medicine, Oregon Health and Science University, 3181 Sam Jackson Park Road, Portland, OR 97239-3098 USA; VA Portland Health Care System (VAPORHCS), 3710 SW US Veterans Hospital Rd, Portland, 97239-9264 OR USA

**Keywords:** Turning, Parkinson’s disease, Dynamic stability

## Abstract

**Background:**

The ability to turn while walking is essential for daily living activities. Turning is slower and more steps are required to complete a turn in people with Parkinson’s disease (PD) compared to control subjects but it is unclear whether this altered strategy is pathological or compensatory. The aim of our study is to characterize the dynamics of postural stability during continuous series of turns while walking at various speeds in subjects with PD compared to control subjects. We hypothesize that people with PD slow their turns to compensate for impaired postural stability.

**Method:**

Motion analysis was used to compare gait kinematics between 12 subjects with PD in their ON state and 19 control subjects while walking continuously on a route composed of short, straight paths interspersed with eleven right and left turns between 30 and 180°. We asked subjects to perform the route at three different speeds: preferred, faster, and slower. Features describing gait spatio-temporal parameters and turning characteristics were extracted from marker trajectories. In addition, to quantify dynamic stability during turns we calculated the distance between the lateral edge of the base of support and the body center of mass, as well as the extrapolated body center of mass.

**Results:**

Subjects with PD had slower turns and did not widen the distance between their feet for turning, compared to control subjects. Subjects with PD tended to cut short their turns compared to control subjects, resulting in a shorter walking path. Dynamic stability was smaller in the PD, compared to the healthy group, particularly for fast turning angles of 90°.

**Conclusions:**

The slower turning speeds and larger turning angles in people with PD might reflect a compensatory strategy to prevent dynamic postural instability given their narrow base of support.

## Background

An important component of mobility is changing directions while walking. The ability to modify the locomotor trajectory is a fundamental, but complex, component of walking behavior. Turning requires the central nervous system to coordinate body re-orientation towards a new travel direction, while continuing with the on-going step cycle, and maintaining postural stability in the medial-lateral plane [1]. Falls during turning are particularly dangerous because they often result in a lateral fall, which results in an eight-fold increase in hip fractures compared with falls during straight-ahead walking [2–4].

Difficulty in turning is a common complaint in people with Parkinson’s disease (PD) [5–7]. Laboratory studies of turning in people with PD have reported reduced speed, increased turning duration, increased number of steps [6, 8, 9], a narrower base of support [10], and impaired segmental coordination of rotation (“en-bloc”) [11–14].

However, objective quantification of dynamic stability during turning and modification of turning strategies across different turning angles and gait velocities have not yet been explored. Dynamic stability, i.e. the ability to control the body’s center of mass (COM) over its moving base of support, is compromised during a turn since the COM may momentarily move outside the base of support naturally, increasing the risk for falls [15]. Most studies have primarily focused on the analysis of a single turning task executed at preferred speed over a single turning angle, or have analyzed on-the-spot turns [8, 16–18]. However, a single turning task does not reflect real-life situations, which often require sequential, quick, and unpredictable turns [18].

Turning is more likely vulnerable to functional impairments than straight ahead gait since turning involves more inter-limb coordination, more coupling between posture and gait, and modification of locomotor patterns requiring frontal lobe cognitive and executive function that play a role in postural transitions [19, 20]. Given the well-documented problems of falls and turning deficits in people with PD, it is important to carefully consider how we currently evaluate their turning ability and to investigate their dynamic stability across a broader range of turns. The purpose of this study was to determine if people with PD have speed-specific and/or angle-specific impaired dynamic stability during turning in a complex locomotor task that emulates real-life mobility challenges.

## Methods

### Subjects

Twelve PD subjects (66.3 ± 6 years, four females) and 19 control subjects (67 ± 8 years, seven females) participated in the study. PD and control subjects with other causes of balance impairment (including somatosensory, visual, vestibular, or orthopedic disorders) were excluded. All subjects with PD had mild to moderate PD (Hoen&Yahr 2–3) and were tested in the morning in their ON levodopa state, one hour after their last dose. Immediately before each experimental session, the PD subjects were scored with the Motor Subsection of the Unified Parkinson's Disease Rating Scale (UPDRS). The mean UPDRS III score was 23.8 ± 8 and the Postural Instability and Gait Disability (PIGD) score was 1.7 ± 1.4. None of the subjects with PD experienced freezing of gait during testing. All subjects gave informed consent according to the Declaration of Helsinki, and the Institutional Review Board of OHSU approved the study.

### Experimental procedure and data collection

**Fig. 1:**
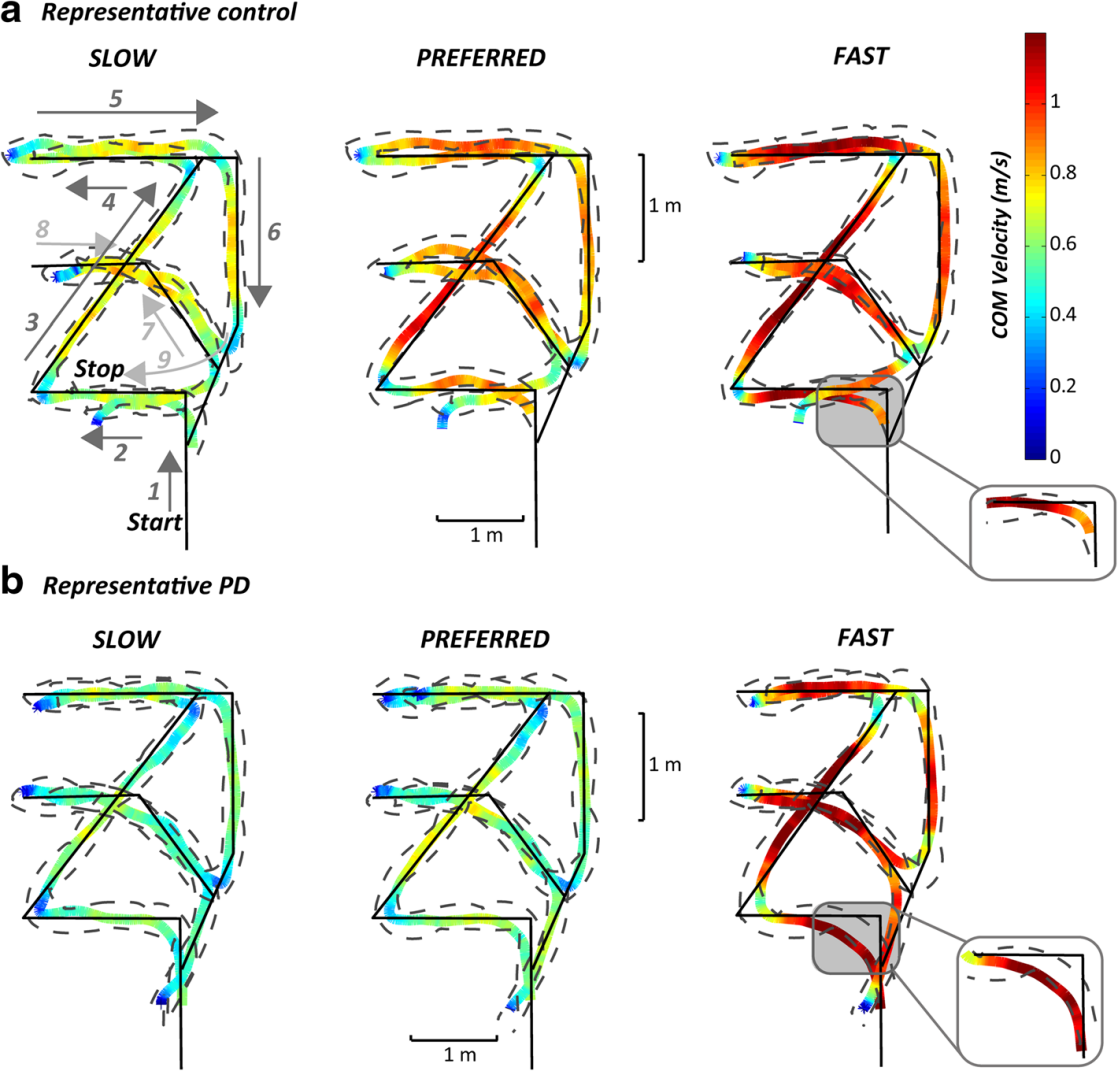
Representative examples of CoM and ankle trajectories for the three requested speeds of trial execution: slow, preferred, and fast. The COM trajectory and its velocity (color code), together with the right and left ankle trajectory (dashed) for a representative control subject **a** and a subject with PD **b**. Solid black line represents the path reference traced on the floor with direction of walking indicated by arrows and numbers. The box insert for the fast trajectory shows a zoomed 90° turn trajectory in which the subject with PD has the COM outside the base of support longer than the control subject

Subjects were instructed to walk following a path traced on the floor with tape, which was composed of a route mixed with short straight paths interspersed within eleven turns ranging from 30 to 180° (see Fig. 1). Specifically, the planned sequence of turns was: 1) 90° left, 2) 135° right, 3) 135° left, 4) 180°, 5) 90° right, 6) 30° right, 7) 165° right, 8) 45° left, 9) 180°, 10) 45° right, 11) 135° right. Here we report results about seven of these turns, only: 180°, and left and right turns of 90 and 135°. We focused on such turns because they were always consistently taken, while other turns, due to the design of the route, often induced subjects to avoid them (45°) or to merge two consecutive turns into a single turn (30 and 165°). Subjects were asked to perform the route 12 times at three different speeds: four at their comfortable (preferred) speed, four at faster, and four at slower speeds. To calculate body kinematics and COM trajectories, subjects wore a set of 15 reflective markers placed bilaterally on the fifth metatarsophalangeal joints (5MTJ), lateral malleolus, knees, shoulders, anterior- and posterior-superior iliac spines, and anterior to ears. Body kinematics (acquired with a 8-camera Motion Analysis system, Santa Rosa, CA; sample rate 60 Hz) and anthropometric tables [21, 22] were used to estimate the instantaneous position of the COM in the sagittal, frontal and transverse planes as a weighted sum of all segments’ COM positions. COM velocity was calculated as the first derivative of the COM position. Figure 1 illustrates the route superimposed on the body COM and footfall trajectories in two representative subjects (one control and one PD subject) walking at slow, comfortable, and fast speeds.

### Data analysis and extracted parameters

*Turning onset and offset* were identified from pelvis rotation in the transverse plane (i.e., rotation around the vertical axis or yaw). The four markers placed on the anterior- and posterior-superior iliac spines were used to compute pelvis orientation and the three Tait–Bryan angles were estimated with the 2D Singular Value Decomposition method [23, 24]; pelvis angles were related to the first acquisition frame in which subjects were standing with their feet on the starting line. Pelvis yaw was then filtered, with a fourth-order zero-lag Butterworth filter with a cut-off frequency of 1.5 Hz, and this signal was used to identify turn onset and offset. Specifically, we defined a turn as a section of the walking trajectory when pelvis yaw rate exceeded 30°/s and movement duration was longer than 0.5 s.

*Dynamic stability* during turning was computed as a function of the body COM position and velocity in the transverse plane relative to the lateral edge of the foot support. Dynamic stability was measured as an estimate of the Extrapolated Center of Mass coordinates (ECOM) adapted from McAndrew Young et al. [25] which was calculated as follows:

$$ X{E}_{COM}(t)={X}_{COM}(t)+\frac{{\overset{.}{X}}_{COM}(t)}{\omega_0} $$

$$ Y{E}_{COM}(t)={Y}_{COM}(t)+\frac{{\overset{.}{Y}}_{COM}(t)}{\omega_0} $$

where *X*_*COM*_ and Y_*COM*_ are the coordinates of the COM on the horizontal plane, ${\overset{.}{X}}_{COM}$ and ${\overset{.}{Y}}_{COM}$ are the components of the COM velocity on the horizontal plane, EX_*COM*_ and EY_*COM*_ are the coordinates of the ECOM on the transverse plane, and ω_0_ represents the eigenfrequency of an inverted pendulum and is computed as

$$ {\upomega}_0=\sqrt{\frac{g}{l}} $$

where *g* (9.81 m/s^2^) is the gravitational constant and *l* is the mean distance between the COM and the outside border of the foot in the horizontal plane measured in the trial.

**Fig. 2:**
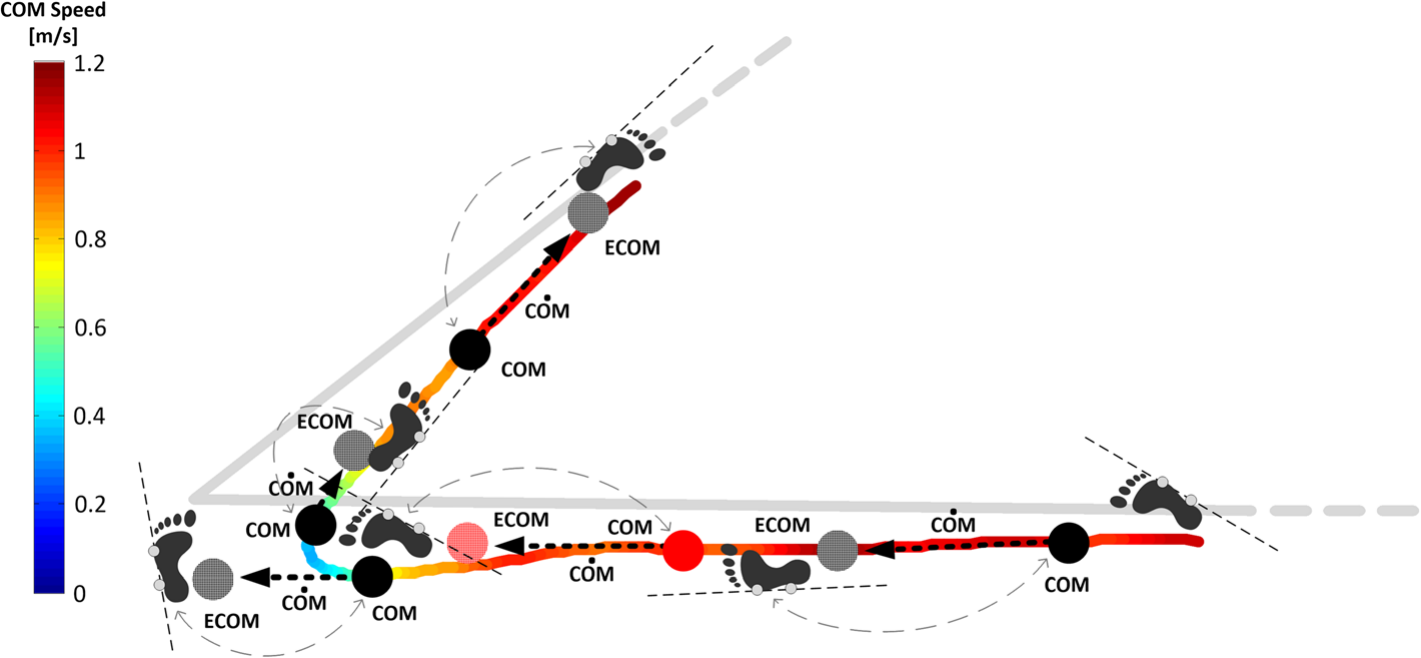
Example illustrating trajectory of the COM and ECOM during first 135° turn for a subject with PD at fast speed. Thick grey line represents the path reference traced on the floor. Footprints are aligned to the line joining the marker on the ankle and the marker on the fifth metatarsophalangeal joint; the area between the line on the left foot and the line of the right foot defines the lateral margins of stability. Specific COM and ECOM positions are represented with a black and grey circle, respectively. The black dotted arrow joining the COM and the ECOM represents the COM velocity vector. When the COM or the ECOM is outside the lateral margin of stability, it is represented in red or light red, respectively. Footprints, COM position, ECOM position, and the direction of the COM velocity vector are represented at the time instant of the heel strike; grey dotted double arrows link the COM position to the foot that is hitting the ground. It is interesting to see that the COM and the ECOM were found to be outside the lateral margin of stability at the onset of the turn

Steps were identified by the trajectories of the markers on the lateral malleolus. The following measures were computed on the whole route:

- *Total length of the COM trajectory*: the length of the COM trajectory on the transverse plane over the whole route (to look for cutting of corners);
- *Total duration*: the time taken to complete the route;
- *Mean velocity of the trial*: the total length of the COM trajectory divided by the total duration;
- *Mean step length* : mean distance between two foot-contacts;
- *Coefficient of variation of step length*;
- *Total number of steps*;
- *Mean step duration*: mean value of the step duration defined as the time between two foot-contacts;
- *Coefficient of variation of step duration*;
- *Mean double support time*: mean value of the time interval in which both feet were on the ground, expressed as a percentage of gait cycle duration;

The following measures were computed for each turn:

- *Turning duration*: the time between onset and offset of a turn;
- *Dynamic stability during turns*: the percentage of time during turning in which the COM (and ECOM) were outside the lateral Margin of Stability (MOS), and the distance between the COM (or ECOM) and the lateral MOS. Lateral MOS was defined as the planar region between the lines through the markers on the lateral malleolus and 5MTJ of the left and right feet. A graphical representation of these variables is illustrated in Fig. 2.

**Fig. 3:**
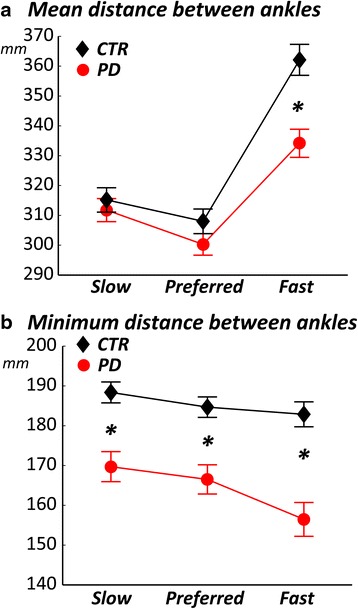
Minimum distance between the ankles is smaller in subjects with PD than control subjects. Comparison of mean **a** and minimum **b** distance between the ankles in healthy and PD subjects across the 3 requested speeds of trial execution. Group means (±SEM) are reported. *t*-test *p*-value: *:*p* < 0.05

### Statistical analysis

Normality of the data was verified by the Shapiro Wilk test before parametric analyses were performed. A 2x3 repeated measures ANOVA, group (CTR vs PD) x speed of execution (slow, preferred, and fast) was performed to investigate the effects of group and intended speed, as well as their interactions. To control for actual, rather than intended, speeds, we repeated the analyses with trials grouped, this time, by their actual speed. The actual mean velocity of execution of each trial was used to match trials of subjects with PD and control subjects. In this secondary analysis, regardless of the intended speed, a trial has been assigned to the low (<50 cm/s), medium (50–65 cm/s), or high (>65 cm/s) velocity group depending on the actual average velocity of the trial. Significant effects were subjected to post-hoc Student’s t-tests and Bonferroni corrected for multiple comparisons. Statistical analyses were performed using NCSS (Kaysville, UT).

## Results

The COM trajectory and its velocity, together with the foot trajectories of a control subject and a representative subject with PD walking on the mixed route are illustrated in Fig. 1. These examples confirmed the typical slow turning and walking of subjects with PD compared to control subjects. In addition, PD subjects were less accurate than control subjects since they cut short the angle’s corner of the path, resulting in less sharp actual turns.

Consistent with this qualitative observation, total length of the COM walking trajectory was significantly shorter in subjects with PD at all speeds (Table 1).

**Table 1:** Summary of the spatio-temporal gait parameters recorded during the mixed route path in healthy controls (CTR) and subjects with PD, grouped by intended velocity of execution (these metrics are calculated including both gait and turning, except for the Mean turning duration). Results of the Repeated Measure ANOVA comparing trials grouped by intended and matched speed are reported in the far-right columns. Statistically significant differences are bolded

Route path		*Slow*		*Preferred*		*Fast*			*Intended speed*			*Matched speed*		
*Measure*		*Mean*	*STD*	*Mean*	*STD*	*Mean*	*STD*		*Group*	*Velocity*	*Interaction*	*Group*	*Velocity*	*Interaction*
*Total length of the COM trajectory (m)*	***CTR***	17.5	1.2	17.3	0.9	15.2	1.1	***F-value***	***9.18***	***76***	0.3	***18.55***	***19.9***	1.6
	***PD***	16.3	1.0	16.3	1.1	14.3	1.2	*p-value*	***0.008***	***<0.00001***	0.7	***0.0002***	***<0.00001***	0.2
*Total duration* *(s)*	***CTR***	37.3	7.0	33.0	4.7	21.9	4.1	***F-value***	0.23	***58***	0.6	2.4	***396***	**6.5**
	***PD***	34.7	8.9	32.9	6.5	22.0	3.2	*p-value*	0.6	***<0.00001***	0.6	0.13	***<0.00001***	**0.004**
*Mean velocity of the trial (m/s)*	***CTR***	0.49	0.07	0.53	0.07	0.7	0.09	***F-value***	0.7	***56***	2	0.3	***786***	**11.4**
	***PD***	0.50	0.1	0.51	0.09	0.6	0.1	*p-value*	0.4	***<0.00001***	0.1	0.6	***<0.00001***	**0.0002**
*Mean turning* *duration (s)*	***CTR***	1.5	0.02	1.4	0.01	1.5	0.01	***F-value***	4.1	***6.2***	0.02	0.6	1	0.9
	***PD***	1.6	0.01	1.5	0.12	1.6	0.01	*p-value*	0.06	***0.006***	1	0.4	0.4	0.4
*Mean step length (m)*	***CTR***	0.42	0.04	0.42	0.04	0.48	0.05	***F-value***	2.8	***26***	0.2	***12***	***15.3***	0.68
	***PD***	0.39	0.06	0.39	0.07	0.45	0.08	*p-value*	0.1	***<0.00001***	0.8	***0.002***	***<0.00001***	0.51
*CoV step length*	***CTR***	0.27	0.04	0.28	0.03	0.30	0.04	***F-value***	***9.5***	***4.6***	0.3	***10.9***	1.96	0.07
	***PD***	0.31	0.09	0.34	0.08	0.34	0.06	*p-value*	***0.008***	***0.02***	0.7	***0.003***	0.15	0.93
*Total number of steps*	***CTR***	59	16	56	7	46	7	***F-value***	0.5	***13***	0.3	1.15	***11.5***	0.08
	***PD***	55	17	55	13	44	11	*p-value*	0.5	***0.0001***	0.8	0.29	***0.0002***	0.92
*Mean step duration (s)*	***CTR***	0.64	0.09	0.60	0.05	0.49	0.08	***F-value***	0.03	***46***	0.3	0.14	***19.5***	1.14
	***PD***	0.64	0.13	0.60	0.08	0.50	0.07	*p-value*	0.9	***<0.00001***	0.8	0.71	***<0.00001***	0.33
*CoV step duration*	***CTR***	0.38	0.12	0.36	0.05	0.34	0.07	***F-value***	*10*	0.3	1.9	***6.15***	0.26	1.09
	***PD***	0.29	0.08	0.29	0.07	0.33	0.06	*p-value*	***0.007***	0.7	0.2	***0.02***	0.7	0.35
*DS time (% gait cycle)*	***CTR***	18.0	8.3	16.6	4.4	16.3	3.6	***F-value***	0.05	0.5	1.5	0.06	0.84	0.03
	***PD***	16.2	5.4	15.9	3.1	18.0	3.6	*p-value*	0.8	0.6	0.2	0.81	0.44	0.97

*Abbreviations*: *COM* center of mass, *CoV* coefficient of variation, *DS* double support

In addition, several other measures of turning characteristics showed significant differences between subjects with PD and control subjects (Table 1). In particular, the mean turning duration showed a significant group and velocity effect, with turns that were significantly longer in duration in subjects with PD compared to healthy subjects. However, both groups shortened turning duration from preferred to fast requested velocity. Thus, we compared walking and turning metrics both at intended route navigation velocities and matched velocities.

Table 1 compares gait and turning characteristics (and ANOVA results) between subjects with PD and control subjects at both their intended and matched velocities.

Although turning duration was significantly longer in subjects with PD, the total route duration, the mean velocity of the whole trial, and the total number of steps were not different between the groups. Both groups were able to scale these parameters when asked to change their speed, although subjects with PD scaled turning speed less than control subjects between the slow and preferred speeds. Mean step length and mean step duration were sensitive to walking speed while step length CoV and step duration CoV were sensitive to groups. Subjects with PD had shorter steps, longer step duration and larger variability of step length and duration than control subjects. Surprisingly, the double support time (DS) was similar between groups and across speeds.

Similar results still hold when matching for actual (mean) speed of navigating the path, except for mean turning duration that didn’t show group, nor actual, execution velocity effects (Table 1).

### Subjects with PD show a narrow base of support during turns

The *minimum* distance between the ankles during turns was significantly different between groups, (*F* = 10, *p* = 0.007), and across velocities (*F* = 3.3, *p* = 0.05), see Fig. 3. Specifically, the minimum distance between the two feet was significantly shorter in PD, compared to healthy subjects, at slow, preferred and fast speeds. These smaller distances between the feet in subjects with PD still hold when matching for actual speed. However, the *mean* distance between the ankles while turning was significantly different across velocities (*F* = 25, *p* < <0.001) with an interaction effect (*F* = 5, *p* = 0.02), but not between groups (group effect *F* = 3, *p* = 0.1), see Fig. 3.

### PD subjects are unstable during fast turns

Dynamic stability, that is, the mean distance between the COM or ECOM and the lateral margin of the feet was significantly smaller in subjects with PD (group effect: *F* = 7.2, *p* = 0.02, condition effect: *F* = 1.56, *p* = 0.2). However, the percentage of time in which the ECOM was outside the lateral MOS was similar in the two groups and was not affected by requested speed of execution (group effect: *F* = 0, *p* = 1, condition effect: *F* = 16, *p* < <0.0001). In addition, the percentage of time during turning in which the COM was outside the lateral MOS was similar in PD and control groups (group effect: *F* = 0, *p* = 1, condition effect: *F* = 16, *p* < <0.0001) across the three different requested speeds. Similarly, the distance between the COM and the lateral foot margin (when outside the lateral MOS) did not differ between groups (group effect: *F* = 0, *p* = 1, condition effect: *F* = 24, *p* < <0.0001).

**Fig. 4:**
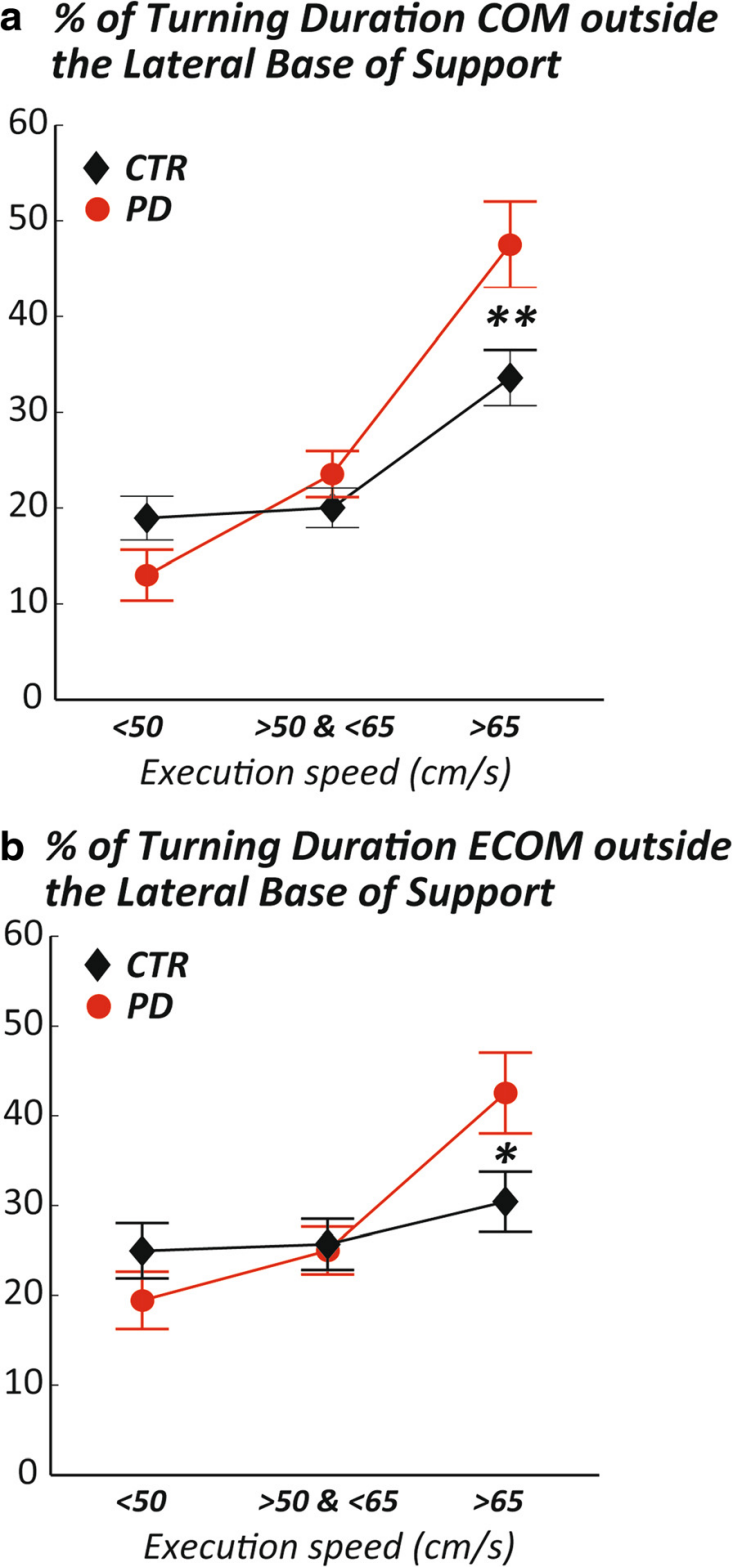
Subjects with PD have their body COM and ECOM outside their base of support for longer duration during fast turns. Group means (±SEM) for the % of turning duration in which COM **a** or ECOM **b** fall outside the lateral base of support in 90 ° turns and matched speeds. *p*-value: *:*p* < 0.05; **:*p* < 0.01

However, these results do not take into account the fact that subjects with PD completed the task at slower speeds compared to healthy subjects, nor the differences that might arise from turning at different angles. For these reasons, we looked at the same measures but matching for actual speed of navigating the path, and selecting specific turns within the path. Selected turns were 90, 135, and 180° because these turns were always performed with a consistent pattern within and between trials. Figure 4 shows that, at matched speed, subjects with PD significantly spend more time with the COM (or ECOM) outside the lateral MOS during turning 90° at fast matched speeds (>65 cm/s) compared to control subjects.

### Subjects with PD complete turns less accurately than healthy subjects

**Fig. 5:**
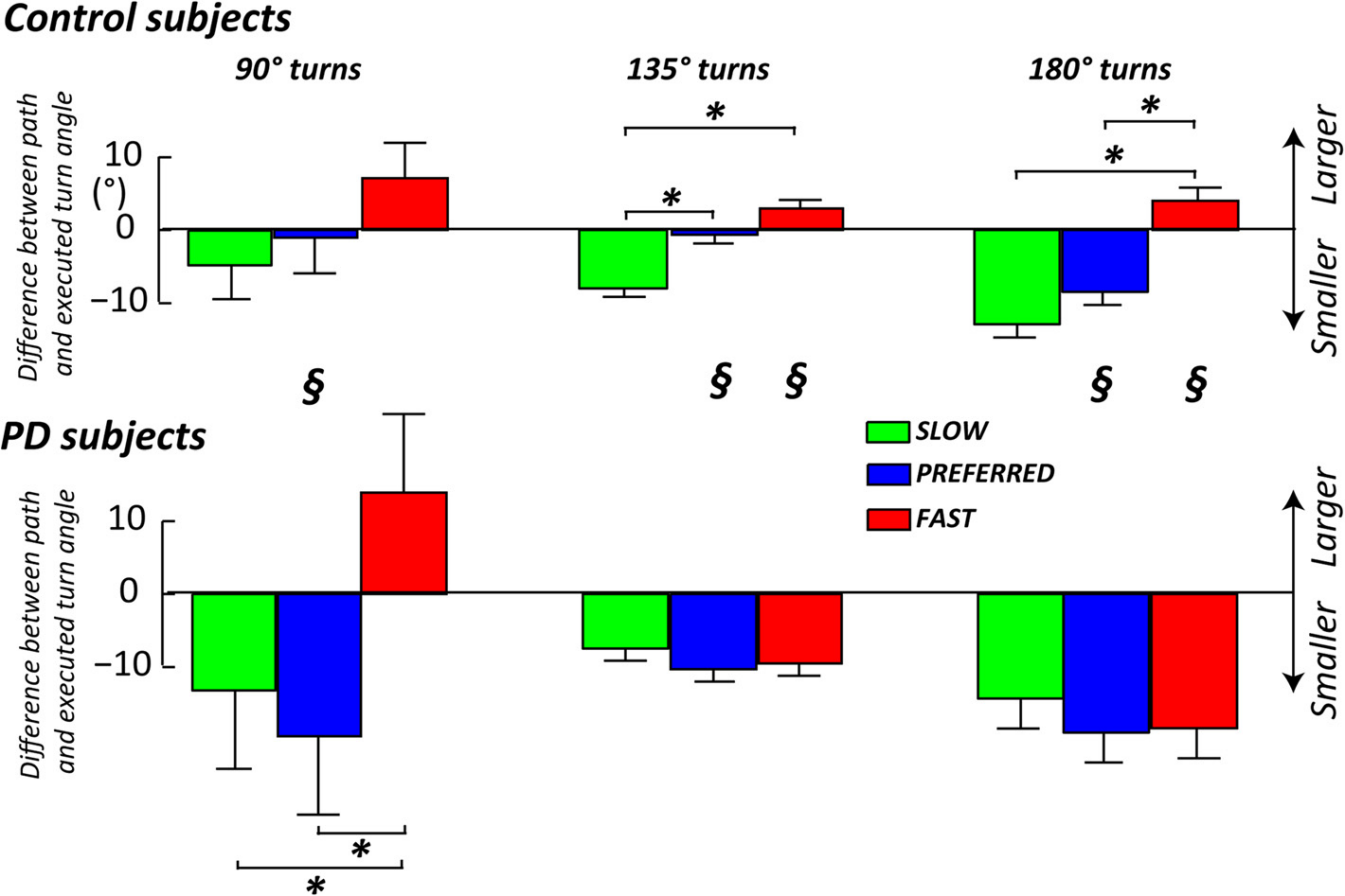
Difference between the path and executed angles was larger for PD than control subjects and did not change similar for the 2 groups across the requested speeds. Group means (±SEM) for the difference between the path and actual, executed turn angles, for the three different requested speeds of execution (grouped by angles of 90, 135, and 180°). 2 × 3 repeated measures ANOVA, group x speed of execution: * = significant difference between different speeds of execution; § = significant difference between groups

Figure 1 qualitatively showed that turning trajectories of subjects with PD were less accurate (insert illustrates cut angles’ corners) compared to healthy subjects. Therefore, we calculated the accuracy of the executed turning angle compared to the one that was marked in the ground. Figure 5 shows how PD subjects tended to turn less sharply than the prescribed floor path (reflected by more negative values in Fig. 5). PD subjects rounded off the turns for all the turning angles and execution speeds compared to control subjects, except for the 90° turn at their fast speed, in which PD subjects tended to exceed (positive value in Fig. 5) the turning angle, similar to control subjects. In fact, healthy subjects showed a trend to turn with larger angles than the path at slow speeds but with smaller turning angles than the path at fast speeds. That is to say they were most accurate at preferred speeds. This trend to modify turning angle for different requested execution speeds was not present in subjects with PD.

## Discussion

The findings of this study demonstrate specific deficits in dynamic stability during turning, but only for specific types of turns, in a cohort of subjects with moderate PD, ON medication. We found the largest postural instability when subjects with PD were asked to perform 90° turns and when they were asked to walk and turn faster than their preferred speed.

Surprisingly, subjects with PD were able to complete the mixed route at a similar, overall duration (and therefore velocity), as healthy subjects. Nevertheless, PD subjects had slower turns, indicating a specific deficit while turning, which was compensate for by faster than normal straight walking. This is consistent with our previous study showing that patients with early, untreated PD can have normal duration of the Timed Up and Go test even though the 180° turn within the test was significantly slower than normal [26].

Consistent with previous findings of studies that investigated single turns [10], subjects with PD showed a narrower base of support than control subjects while turning at their self-selected speeds. In fact, the minimum distance between the ankles while turning was always shorter in subjects with PD compared to healthy control subjects and the mean distance was shorter at high and low speed but similar to control subjects at medium speed. The narrower base of support during turns resulted in worse dynamic stability, that is, the mean distance between the COM or ECOM and the lateral margin of the foot was significantly smaller in the PD, compared to the control group.

However, when matching for actual execution speed and discriminating between the different turning angles, we found that subjects with PD were more unstable (measured by the percentage of time with the COM or ECOM outside the lateral MOS) than control subjects only during 90° turns at high speeds, (Fig. 4). This finding suggests that sudden changes of direction at 90° to be performed faster than the preferred speed (e.g. such as when rushing in or out of a room or turning quickly in the kitchen) seem to be more problematic than other turns and speeds (e.g. 135 and 180° at medium or low speeds).

Interestingly, subjects with PD were less accurate than healthy subjects in the turning execution (see Fig. 5). Specifically, subjects with PD showed a general tendency to underestimate turning angles compared to control subjects at all velocities and for all turning angles, (except turning angles of 90° at fast speed, in which they executed a path similar to control subjects and were most unstable). In addition, subjects with PD did not scale from underestimating to overestimating execution of turning angles when increasing speed, like control subjects. This result, in light of basal ganglia dysfunction, seems to be consistent with the hypothesis of Morris, Iansek et al. [12, 27] of normal motor commands, but impaired ability to scale movement amplitude in Parkinson’s disease.

The findings presented in this study have very practical implications. The most unstable turns occurred at 90°, which is the most common turning angle made during daily activities [28]. Thus, fall prevention rehabilitation could focus on modifying turning strategies, particularly for 90° turns, such as practicing a wider arc turn, turning more slowly or practicing sustaining a wider base of support. Scaling turning speeds for various speeds of walking is another important characteristic to train in order to reduce falls. People with PD may lose their balance due to inability to change turning strategy with change in motor commands for a change in speed. On the other hand, we speculate that subjects with PD might be actively slowing and widening their turns to compensate for postural instability due to narrower base of support but more evidence is needed to support this hypothesis.

A limitation of this study is that we did not characterize multisegmental coordination, which is known to be abnormal in people with PD. The ‘en-bloc’ turning without sequential top-down rotation of segments could be contributing to their dynamic postural instability when attempting fast, 90° turns. However, previous studies have not characterized multisegmental coordination during continuous, mixed route turning or compared coordination for a variety of size and speeds of turning. It will also be important to evaluate the effects of PD on turning characteristics during unprescribed turns, rather than when following a prescribed path that provided external visual cues, known to be helpful for movement in PD [29–31]. This study also did not investigate the role of levodopa on postural stability during turns so future studies should compare turning characteristics in the ON versus OFF levodopa state.

## Conclusions

Subjects with PD may be actively slowing and widening their turns (cutting corners) and they appear to prioritize stability over speed or accuracy during turning, except for 90° turning angles, where they spent a higher percentage of time with ECOM outside the BOS. Therefore, it may be helpful to train subjects with PD to avoid fast directional changes or to use more compensatory strategies, such as consciously widening their MOS and focusing on accuracy. Lastly, 90° turns, which are among the most common turning angles during daily activities [28], were the most unstable.
